# The mixed blessing of living together or close by: Parent–child relationship quality and life satisfaction of older adults in China

**DOI:** 10.4054/demres.2021.44.24

**Published:** 2021-03-23

**Authors:** Feinian Chen, Ke Shen, Hangqing Ruan

**Affiliations:** 1 Department of Sociology, Maryland Population Research Center, University of Maryland, College Park, USA.; 2 School of Social Development and Public Policy, Fudan University, Shanghai, China.; 3 Department of Sociology, Maryland Population Research Center, University of Maryland, College Park, USA.

## Abstract

**BACKGROUND:**

Geographic proximity between parents and children is increasingly recognized as an alternative measure to coresidence as a gauge for intergenerational support in China. The quality of intergenerational relationships is another important dimension of intergenerational ties that is often underexplored.

**OBJECTIVE:**

We examine the association between parent–child proximity and life satisfaction of older adults and how it interacts with the quality of intergenerational relationships, particularly for vulnerable subpopulations.

**METHODS:**

We use data from the China Longitudinal Aging Social Survey (CLASS 2014). We use ordered logit models to predict life satisfaction scores (ranged 1 to 4).

**RESULTS:**

Our analyses show that parent–child relationship quality is strongly associated with life satisfaction, regardless of living proximity, in our full sample analysis. For those who have a lower-quality relationship with their children, coresidence or close-distance living does not enhance life satisfaction and they indeed have lower life satisfaction than those parents who have all children living farther away but maintain a high-quality relationship with them. At the same time, for those who have a high-quality relationship with their children, we find that close proximity provides added benefits for the subpopulations who are socioeconomically or physically disadvantaged, that is, female, urban, unmarried, and disabled (in terms of being capable of activities of daily living, or ADL) older adults.

**CONCLUSIONS:**

We recognize the interdependence of the quality of the intergenerational ties and parent–child proximity in promoting life satisfaction, particularly for subpopulations who are in stronger need of support from adult children.

**CONTRIBUTION:**

Our study clearly points to the importance of extending the research on intergenerational relationship beyond the boundary of the household and of paying close attention to the affective dimension of intergenerational ties.

## 1. Introduction

*Continuity* and *change* are the two key words often used to describe the current state of East Asian families (Raymo et al. 2015). Against the backdrop of rapid socioeconomic changes, globalization, and the rise of individualism, some aspects of family organization and behavior are remarkably stable. Among them, one persistent feature of East Asian families is the prevalence of multigenerational families and the strength of intergenerational ties. Although intergenerational coresidence in East Asian societies has declined steadily (Yasuda et al. 2011), a convergence toward a conjugal family system has yet to take place in mainland China, Japan, South Korea, or Taiwan, particularly from the perspective of aging parents (Emery, Dykstra, and Djundeva 2019; Lin and Yi 2013; Qi 2016; Raymo et al. 2015; Taniguchi and Kaufman 2017; Whyte 2005; Yasuda et al. 2011).

Based on China’s recent four censuses in 1982, 1990, 2000, and 2010, the multigenerational family has stabilized at about 18% of all types of households (Hu and Peng 2014). The share averaged at 14% and 13% for Africa and Asia, respectively, and was merely 2% for Europe in 2010 (United Nations 2017).^4^ China, in the face of accelerated population aging and inadequate social safety nets, offers a distinctive social and cultural context to revisit the implications of traditional family structures. Rapid economic development and the one-child policy since the 1980s has further reduced fertility and mortality in China and has accelerated the aging of its population to one of the fastest rates ever recorded (Chen and Liu 2009; Wang 2011). The share of the Chinese population aged 65 and over rose from 6.8% in 2000 to 9.3% in 2015 and is projected to almost triple to 26.1% by 2050, outpacing the United States, Canada, United Kingdom, Sweden, and Australia (United Nations 2019). Moreover, unlike other East Asian societies with a similarly fast pace of aging, China is facing the challenge of “growing old before growing rich” (Phillips and Feng 2015). Despite new efforts to extend the coverage of social welfare programs since the early 2000s, both public pension and health insurance systems remain highly fragmented, with rural and urban residents without formal employment entitled to limited social security (Shen, Feng, and Cai 2018). Hence, these multiple challenges in China put mounting pressure on the traditional family support system as a key source of old-age support.

Intergenerational coresidence is generally deemed as a structural manifestation of filial norms and family support in China. However, despite it being the traditionally dominant living arrangement, whether coresidence with adult children promotes well-being for older adults remains a hotly debated question. Empirical studies produce mixed findings, with some reporting beneficial and others suggesting detrimental effects (Chen and Liu 2009; Do and Malhotra 2012; Gee 2000; Sereny 2011; Silverstein, Cong, and Li 2006a). Existing research overwhelmingly focuses on the structural context of family support, that is, living arrangement or household structure, leaving two key issues largely underexplored. First, scant attention is paid to within-family dynamics or heterogeneity among extended families, often due to the lack of such data in household surveys. Not all of these families are alike in their circumstances, and not all parents desire to coreside with their children (Sereny 2011). Researchers often note that while the household provides members with crucial support and integration, coresidence with adult children could also create tension due to the stress of caregiving, unpleasant interactions, or loss of independence (Guo et al. 2016; Hansen and Slagsvold 2015; Kim et al. 2012; White and Rogers 1997; Yan and Kwok 2011). Second, extended family or coresidence could be too narrow a concept. Studies have documented that living independently does not necessarily mean geographical isolation from children, and non-coresident children often live close by (Knodel and Chayovan 2008; Lei et al. 2015; Whyte 2005). Multiple surveys in China reveal that for both adult children and parents the ideal living arrangement is “a bowl of soup’s distance” (meaning living sufficiently close so that a bowl of hot soup will not get cold when carried from one house to the other) rather than under the same roof, as nearby living maintains frequent contact and mutual support while at the same time assures independence and privacy (Hu and Ye 1991; Ma et al. 1994; Yan 1997; Zhang 2012). Such close-distance living and the frequent economic cooperation and emotional support among family members has also been referred to as “modified extended family” or “network family” in the literature (Bian, Logan, and Bian 1998; Chen 2005; Logan and Bian 1999; Logan, Bian, and Bian 1998; Yan 1998).

In this paper, we plan to extend the existing lines of research, directly addressing the abovementioned issues that are underexplored in the literature. We use a newly available, nationally representative data, the Chinese Longitudinal Aging Social Survey (CLASS). First, we go beyond coresidence by examining the association between parent–child proximity and life satisfaction of older adults. Second, we maintain that the impact of coresidence or close-distance living on life satisfaction is not uniform but depends on the quality of the relationship between parents and adult children, and vice versa. We hypothesize that the extent of an interaction effect could differ by subpopulations, particularly for those who may have a stronger need for support from adult children.

## 2. Coresidence and psychological well-being of older adults

Research on the psychological consequences of intergenerational coresidence for older adults is far from reaching a consensus. Instrumentally, coresidence is a main vehicle for children to offer physical assistance and emotional support for older parents (Teerawichitchainan, Pothisiri, and Long 2015; Wang, Chen, and Han 2014). From a normative perspective, coresidence also conforms to the Confucian tradition of filial piety that historically means absolute deference and respect for one’s parents and ancestors, a dominant moral philosophy in East Asian societies (Chu, Xie, and Yu 2011; Whyte 2003). Considerable evidence lends support to the benefits of coresidence, suggesting that older adults living with children enjoy better mental health and higher life satisfaction than those living alone, particularly in Asian societies. For example, several studies show that living with children is positively associated with older adults’ subjective well-being in mainland China (Chen and Short 2008; Mao and Han 2018), benefits older adults’ emotional health in Vietnam and Thailand (Teerawichitchainan, Pothisiri, and Long 2015), and improves older adults’ life satisfaction in Malaysia (Kooshiar et al. 2012).

However, it is also noted that coresidence could lead to increased family conflict, thus undermining the advantages of mutual support for both generations (Ko 2012; Yang and Chhandler 1992). Negative interactions and tension could coexist with cooperation and love in shared living experiences. Nowadays with declined adherence to filial norms, eroding authority of parents, rising generational differences in values, and increased desire for privacy in the process of radical socioeconomic transformations, the risk of tension among coresident family members might be heightened (Li and Liang 2007; Seeman et al. 2004; Whyte 2005; Zhou and Qian 2008; Zimmer and Kwong 2003). Fieldwork studies in a rural village in northeastern China by Yan (1997) also document the conflicts between married sons or daughters-in-law and aging parents in stem families. Some studies in China even found that older adults living with adult children (especially when the spouse is not present in the household) are more depressed, less happy, and have lower life satisfaction than those living with the spouse only (Ren and Treiman 2015). Another comparative study including Hong Kong, urban mainland China, and Taiwan finds that older adults gained better psychological well-being from social participation and living independently with a spouse than from living with children (Miao and Wu 2016).

The impact of living arrangements on psychological well-being also varies by older adults’ status. For example, some studies show that for widowed Chinese older adults, coresidence with adult children was associated with better psychological well-being compared to living alone, while for those who are married, coresidence did not bring additional benefits (Gee 2000; Wang, Chen, and Han 2014). Similarly, in a study of widowed older women in South Korea, coresidence with an adult child significantly lowers the depression scores (Do and Malhotra 2012). There also exists rural and urban differences. For example, one study from China finds that living with adult children was positively associated with rural elders’ life satisfaction but did not improve life satisfaction for urban elders (Mao and Han 2018). Scholars believe that the traditional pattern of living arrangement is beneficial in rural China because it still represents “the fulfillment of a cultural ideal” (Silverstein, Cong, and Li 2006a). The consequence of living arrangements on psychological well-being also varies by gender. One study from Japan shows that the number of children promoted older female adults’ life satisfaction and reduced depressive symptoms, but there was no such effect for older male adults (Okabayashi and Hougham 2014).

## 3. Intergenerational solidarity and ambivalence framework: Beyond the household and heterogeneity of within-family dynamics

The inconsistency in the empirical findings on the association between coresidence and psychological well-being points to a major gap in the literature, that is, the heterogeneity of extended families is not well accounted for when the focal point of research is limited to the household structure. To advance this line of research, we turn to the intergenerational solidarity and intergenerational ambivalence frameworks.

The intergenerational solidarity framework characterizes the diversity in parent–adult child relationships by characterizing six multifaceted analytical dimensions of solidarity: structural, associational, functional, normative, consensual, and affective solidarity (Bengtson 1975; Roberts, Richards, and Bengtson 1991; Silverstein and Bengtson 1997). Coresidence, as a manifestation of structural solidarity, could facilitate functional solidarity (the giving and receiving of support across generations) as well as associational solidarity (the type and frequency of contact between intergenerational family members), thereby improving the welfare of both generations (Bengtson 2001).

At the same time, it is noteworthy that structural solidarity could transcend the boundary of the household. Geographical proximity could become a more relevant structural concept than coresidence in light of increasing demands for privacy and independence from both generations. Scholars have correctly pointed out that a decline in coresidence should not be equated with a weakening of intergenerational ties (van Gaalen, Dykstra, and Komter 2010; Gruijters 2017; Knodel 2014). Instead, living nearby can easily allow parents and children to maintain a high level of contact and to exchange resources and support (Hermalin, Ofstedal, and Li 1992; Hermalin and Yang 2004). Thus, the psychological benefits associated with coresidence could extend to close-distance living.

Research on how parent–child proximity influences older adults’ well-being, particularly on psychological well-being in the Asian context, has been very limited. Studies in other contexts clearly point to the importance of geographic proximity for intergenerational relationships. For example, child–mother proximity has consequences for the intensity of intergenerational contact and eldercare in Sweden (Holmlund, Rainer, and Siedler 2013), and children living close significantly decrease older parents’ likelihood of moving to a care institution in the Netherlands (Van Der Pers, Kibele, and Mulder 2015). In China, patrilocality and intergenerational proximity still demonstrate a remarkable resilience (Gruijters and Ermisch 2019); non-coresident children often live close to their parents (Chan 2005), contact them frequently, and provide regular help (Bao 2016). Recent statistics from nationally representative datasets such as China Health and Retirement Longitudinal Study and China Household and Finance Survey suggest that living apart but close has become increasingly common, particularly in urban China (Gan and Fong et al. 2020; Lei et al. 2015).

Another drawback of relying on coresidence is that it does not tap into the dimension of affective solidarity, or evaluations family members express about their relationship with other members. It is overly simplistic to assume that everybody lives harmoniously under the same roof or in close distance. Based on the intergenerational solidarity framework, structural solidarity might also be connected with a low level of consensual solidarity (agreement in opinions and values between generations) and/or a low level of normative solidarity (expectations regarding filial obligations and parent obligations), that in turn translate into the deterioration of affective solidarity (Bengtson 2001; Lowenstein 2007). For instance, tensions and conflicts in multigenerational families may arise due to discrepancies in lifestyle and sharing of household facilities, and in-law relationships are even tenser due to disagreements on child-rearing and discipline (Chui 2007). Aging parents might feel disappointed and depressed when their adult children cannot afford to move out of the parental household and achieve the parental expectation of success (Ko 2012; Ward and Spitze 1992). Older adults could also feel overwhelmed by obligations to provide assistance with household chores and grandchild care. Intergenerational ambivalence is thus a useful concept to supplement the intergenerational solidarity framework, reflecting the paradox between conflict and solidarity in intergenerational relationships (Lin et al. 2018; Luescher and Pillemer 1998; Silverstein et al. 2010). A growing body of research underscores the importance of both parent–child proximity and intergenerational relationship to the well-being of older adults; however, they have not considered the intersections of these two dimensions of intergenerational solidarity and how they influence life satisfaction yet (Chai and Jun 2017; Lin, Chang, and Huang 2011; Lowenstein and Katz 2005).

To sum up, a key principle of the intergenerational solidarity and ambivalence framework is that none of the dimensions of intergenerational relationships operate in a vacuum, but rather they are interdependent. The various dimensions of solidarity and ambivalence could coexist depending on family dynamics, both within the household and beyond the boundary of the household. We elaborate this further in the hypotheses section.

## 4. Research hypotheses

Guided by the concepts of intergenerational solidarity and ambivalence, we extend the literature on intergenerational ties and psychological well-being in China in a few major ways. First, in addition to coresidence, we also examine the role of close-distance living, broadening the structural context for the intergenerational exchange to parent–child proximity. We hypothesize that having adult children living nearby may have associations similar to those of coresidence. Second, we include an additional dimension of the intergenerational relationship (i.e., the relationship quality between parents and adult children) into our empirical analysis. We hypothesize that the effect of close-distance living or coresidence may well depend on the emotional bond between the generations. A high-quality relationship could enhance well-being while a lower-quality relationship could have the opposite effect.

We also want to acknowledge potential endogeneity issues upfront. Coresidence with children is not random but is likely to be selective on individual and family characteristics. Determinants of coresidence such as economic resources or care needs are likely to influence well-being. In addition, the migration of adult children away from their parents is likely to be selective as well. For instance, children with little human capital might be left behind and coreside with their parents in the village while the resourceful and highly motivated children could move away and settle down in the cities. Nonetheless, given the strong normative culture of the multigenerational household and the rigidity of the household registration system (*hukou*), existing literature has documented that coresidence is much less associated with family resources in China as compared with some other contexts (Chu, Xie, and Yu 2011; Davis-Friedmann 1991; Zhang 2004). Coresident children and non-coresident children are often not substantially different in socioeconomic characteristics (Zeng and Xie 2014). In order to account for the endogeneity bias, we also control for children’s characteristics such as their education and occupation as well as multiple health indicators of the older adults in the analyses.

In the context of China, we also expect that the association between parent–child proximity and psychological well-being might differ across subpopulations. The urban–rural divide is a well-known feature of Chinese society. Rural older adults are much more dependent on their children for old-age support, given that they are eligible for much less generous pension plans and health insurance schemes as compared with their urban counterparts. Also, older adults living in rural China are more likely to hold on to the traditional Confucian culture and have higher expectations of filial piety than in urban areas (Luo and Zhan 2012). Thus, they could be more sensitive to the negative aspects of intergenerational relations. The same hypotheses apply to women, the widowed, and those with some restriction in activities of daily living (ADL), who are also more vulnerable and more dependent on their children, compared with men, the married, and those fully independent in ADL. For example, there is a vast amount of literature documenting the crucial role of the spouse in old-age support and health protection globally (Bratt, Stenström, and Rennemark 2017; Gyasi and Phillips 2020; Lehane et al. 2018; Tseng, Petrie, and Leon-Gonzalez 2017). The absence of spousal support and health deterioration could mean that the ties with children could play a more prominent role in shaping the well-being of older adults.

## 5. Data and variables

In this study, we use the newly available data from the Chinese Longitudinal Aging Social Survey (CLASS), collected by the Institute on Aging, Renmin University of China in 2014. It is a nationally representative survey, covering 28 provinces, 134 counties, and 462 villages/neighborhoods. It includes 11,511 older adults above the age of 60. A multistage stratified probability sampling method was used in CLASS: county-level areas (including counties, county-level cities, and districts) are the primary sampling units, and village/neighborhood committees are the secondary sampling units. Then a random sample of households is drawn from each village/neighborhood committee, and one elderly person was randomly selected in each household as the survey respondent. The overall age distribution of the CLASS sample is similar to that of China’s latest census in 2010, and data quality is shown to be satisfactory (Du et al. 2016). The survey collects rich information, including demographic and socioeconomic conditions, health and services, pension/retirement planning, cognitive abilities, and attitudes toward aging. One unique feature that distinguishes CLASS from other existing aging surveys in China (such as China Health and Retirement Longitudinal Study and China Longitudinal Healthy Longevity Survey) is that it includes detailed questions on the intergenerational relationship – including proximity, contact, exchange, and relationship quality – with all adult children. As our research focuses on the impact of living proximity and relationship quality with adult children on elders’ psychological well-being, the sample is restricted to older adults who have at least one alive child, and about 0.43% of the sample (N = 50) are excluded in this study. In addition, we also exclude respondents with missing values on key variables (N = 693, 6.02%). Our final sample includes 10,768 older adults, with 6,411 living in urban areas and 4,357 living in rural areas.

Our key dependent variable is the psychological well-being of older adults, as measured by life satisfaction, created from responses to the survey question: “On the whole, how do you feel about your life?” The answers range from “unsatisfied,” “so-so,” and “satisfied” to “very satisfied,” which were given an ordinal variable (1 to 4), with a higher score representing higher satisfaction with life. As seen in Table 1, the overall level of life satisfaction in the sample is high, with a mean of 3.055. There is barely any gender disparity in life satisfaction. As expected, rural residents have a lower level of life satisfaction than their urban counterparts, given the large difference in socioeconomic resources, living conditions, and welfare. The married have a higher average than the unmarried (most likely to be widowed). The ADL-independent older adults have higher life satisfaction than the ADL-disabled adults.

Our unit of analysis is the parent, with one record per older adult in the dataset. We first start with a measure of living proximity to adult children, constructed as a three-category variable: (1) “coresidence with children,” (2) “children living close by,” and (3) “children all living farther away,” with the latter categories excluding classification in previous ones. Coresidence is defined as the older adult living with at least one of his/her children in the same house. Children living close by is defined as the older adult living with at least one of his/her children in the same village or neighborhood but none of the children are living under the same roof. The third category, “children all living farther away,” refers to those who have all of their adult children living in different villages or neighborhoods. These three categories are mutually exclusive. For example, those included in the category of children living close by exclude any possibility that they may live with any of their children in the same house or have all the children living farther away. As seen in Table 2, about 44% of the older adults in the sample live in the same house with at least one of their children. Among 56% of those who do not coreside with their children, one-third of them (18.9% out of 56%) have at least one child living close by, as in the same village or neighborhood. About 37% of the older adults have children all living farther away. If we combine coresidence and close-distance living together, that accounts for the majority of the sample, suggesting a high level of structural solidarity between parents and their adult children.

Relationship quality taps into affective solidarity, which is another dimension of intergenerational solidarity that we feature in this paper. CLASS contains three questions that measure both the positive and negative aspects of parent–child relationship quality. The positive aspect is measured by a question on whether the parent feels emotionally close to the child (close = 1; not close = 0). The negative aspect is measured by two indicators. One is based on a question of whether the parent thinks the child does not care enough about him/her (never = 1; sometimes or often = 0). The other is on whether the older adult considers the child as too demanding in terms of financial support or assistance with grandchild caring or household chores (never = 1; sometimes or often = 0). If the respondent scores 1 in all three aspects with his/her child, we code this as having a high-quality relationship with the child. Otherwise, it is coded as a 0, indicating a lower-quality relationship with the child.

It is important to note that the abovementioned relationship quality variable measures the relationship between a parent and each of his/her child, but a parent on average has 3.13 number of children in the sample. Because our unit of analysis is the parent, not the parent–child dyad, we then need to aggregate the variables to the parent level. Because our key interest in the paper is to test the hypothesis of whether the effect of proximity depends on relationship quality, we construct a set of variables that jointly reflect both characteristics. In essence, we expand the above-described three-category variable (living together, nearby, or with all children farther away) to a six-category variable (parents with all children living farther away and a lower-quality relationship as the reference). We differentiate each living-proximity category by relationship quality (1 = high-quality relationship, 0 = lower-quality). Relationship quality is specific to each proximity category. For example, for parents who live together with an adult child, we take into account the relationship quality with only this coresident child and disregard relationship qualities with any other children. In the case of multiple children in one type of living proximity category, for example, two children living in the same village/neighborhood but not in the same household, we code them as having a high-quality relation if at least one child has a harmonious relation with the parents. We also experiment with a more conservative coding scheme, that is, when both children in such living arrangement have a harmonious relationship with the parents, we code it as a high-quality intergenerational relationship. The results are consistent (results for the more conservative coding scheme not shown).

The distribution of the six-category variable is shown in Table 2. As shown in Table 2, overall, in each living proximity category, the majority of the parents enjoy the high-quality relationship with their adult children; namely, they are emotionally close to their children, and their children care enough about them and are not asking for too much. Close to a quarter of the older adults and their children have a lower-quality relationship, suggesting tension and conflict could be more dominant in these relationships than cohesion and support. Overall, the socially and physically disadvantaged groups – namely women, rural residents, the unmarried (who are most likely to be widowed, given extremely low divorce rates for the older adults), and the ADL-disabled adults – are more likely to live closer to their children than men, urban residents, the married, and the ADL-independent ones.

## 6. Methods

In order to tackle the gaps in the existing literature and adequately address our research questions, the analytical strategies include two steps. As our dependent variable, life satisfaction is a four-category ordinal variable, the ordered logit models are applied. The first step of the analysis provides the baseline for the entire study. We focus on the impact of children’s proximity on life satisfaction. We include a wide range of potentially confounding variables of older adults’ characteristics, such as age, gender, rural/urban residence, marital status, work status, literacy, entitlement to public pension, cognitive function measured by MMSE (mini-mental state examination), and physical health measured by ADL (activities of daily living) and IADL (instrumental activities of daily living). Moreover, we control for their children’s characteristics that would likely influence parents’ well-being, including the number of alive children, whether any of the children received post-secondary education, and whether any of the children holds a position as a manager or professional. Descriptive statistics of these covariates are included in Table 1.

In the second step of the analysis, the key independent variable is changed to the six-category variable incorporating both proximity and relationship quality under the hypothesis that the effect of proximity interacts with that of relationship quality. In the third step, we conduct the same analyses in the subsamples by gender, urban/rural residence, marital status, and ADL status.

## 7. Results

Results from the ordered logit models are presented in Table 3. Model 1 is the reduced model with only parent–child proximity, and Model 2 controls for all the covariates. Interestingly, we observe that older adults who coreside with or live close to children do not differ from those living farther away from all children in the level of life satisfaction (see Model 1). After adjusting for the confounding variables, the results remain robust (Model 2). However, we should not jump to the conclusion that living proximity does not matter for the well-being of older adults. As indicated by the intergenerational solidarity and ambivalence framework, living close to or in the same household with adult children could be a blessing or a curse, depending on whether parents and children have an affectionate and supportive relationship or whether the bond is fraught with conflicts. Without taking the dynamics of the intergenerational relationship into consideration, the positive and negative effects of living proximity could cancel each other out.

The covariates in the models mostly operate in expected directions. For example, older adults who are currently working, entitled to the public pension, and having better cognitive and physical health report higher life satisfaction. There is a curvilinear association between age and life satisfaction. Women are much more satisfied with life than men, while we find little difference between rural and urban residents as well as between the married and unmarried (most likely to be widowed), which suggests that the bivariate difference we observe in Table 1 is accounted for after controlling for other covariates. In terms of children’s characteristics, more children and more successful children (marked by receiving a good education and holding prestigious positions, often perceived as bringing honors to their parents) significantly promote parents’ well-being, consistent with earlier research findings (Gao and Qu 2019; Silverstein, Cong, and Li 2006b).

**Figure 1:**
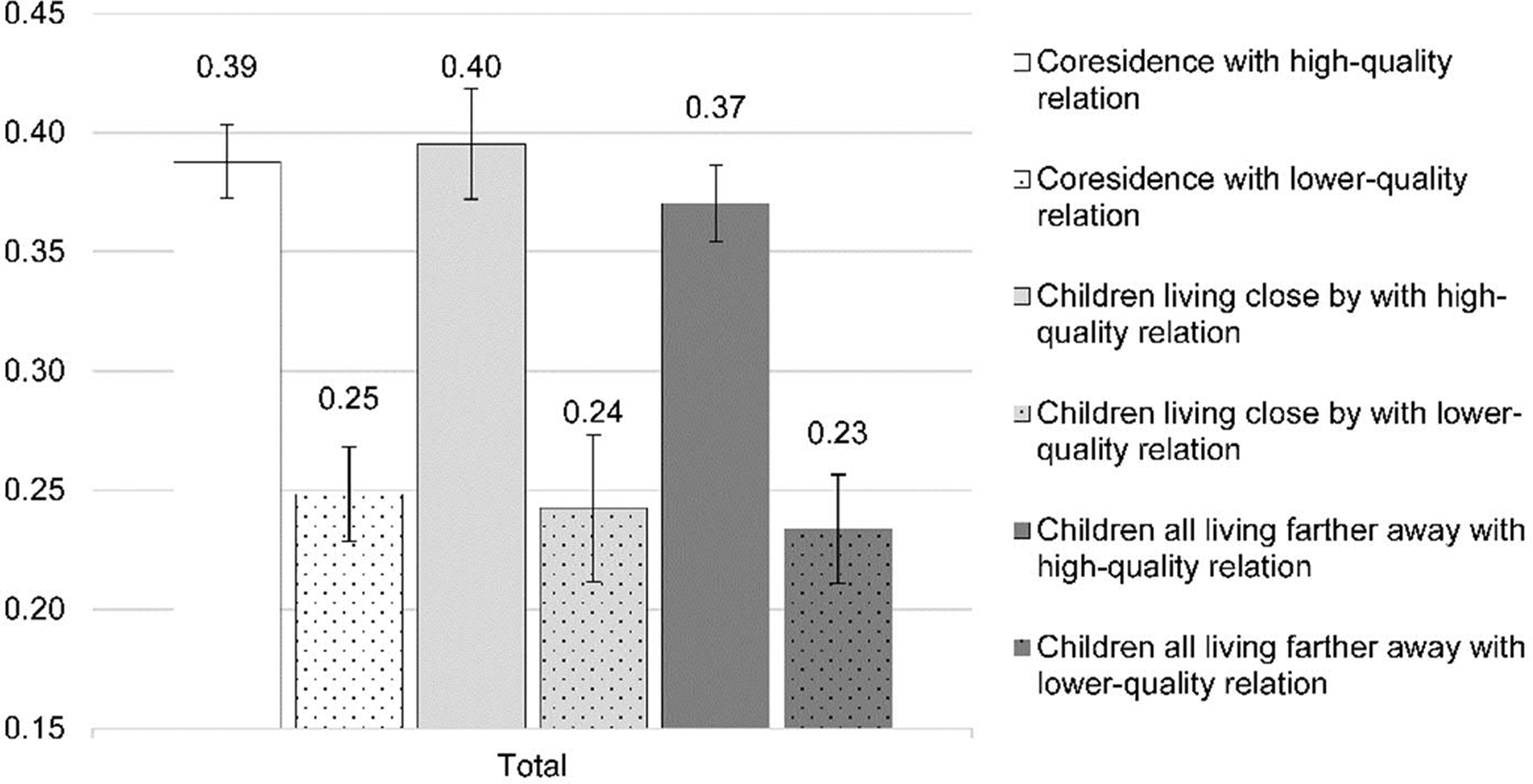
The predicted probabilities of being very satisfied in life by parent–child living proximity and relationship quality (CLASS 2014, N = 10768)

We now move to results from Table 4. In this set of analyses, we examine whether the effect of parent–child proximity interacts with that of intergenerational relationship quality. The results clearly suggest that relationship quality matters, and we see diverging effects of proximity once relationship quality is taken into account. First, when one has a lower relationship quality with his/her children, a proximity to children does not matter, with the reference category being children all living farther away and having a lower-quality relationship. At the same time, better relationship quality translates to higher life satisfaction. To aid interpretation, we present the predicted probability of being “very satisfied” in life by living proximity and relationship quality in Figure 1 (calculation based on coefficients in Table 4, with other covariates held at their means). Regardless of living proximity, those with high-quality relationships enjoy higher life satisfaction, with confidence intervals overlapped across the categories of living proximity. Those with children living close by and having a high-quality relationship have a predicted probability of 0.40 of being very satisfied in life, followed by coresidence (0.39) and all children living farther away categories (0.37), with the confidence intervals overlapped. Figure 1 also shows that those with a lower-quality relationship with children are less likely to have high satisfaction in life in general, with the confidence intervals overlapped across all living proximity categories.

**Figure 2:**
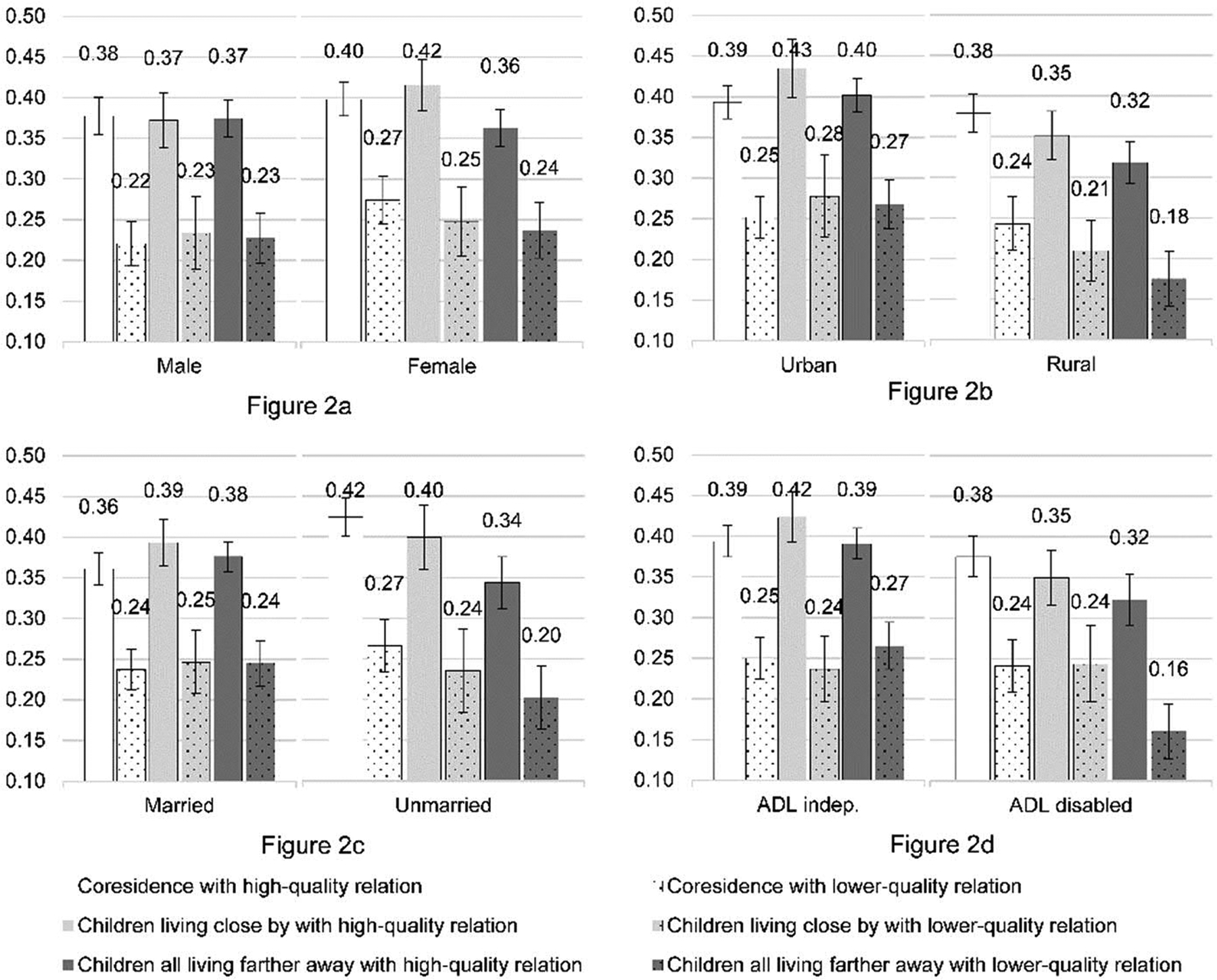
The predicted probabilities of being very satisfied in life by parent–child living proximity and relationship quality in subsamples (CLASS 2014)

The full sample analysis may suggest that relationship quality outweighs living proximity. However, our subsample analysis indicates that living proximity and relationship quality are both important for the vulnerable population. Figure 2 shows the predicted probability of being very satisfied with life by parent–child living proximity and relationship quality in subsamples (based on coefficients in Table 4). For example, women who have children living close by and maintain a high-quality relationship report the highest level of life satisfaction (probability of 0.42 of being very satisfied in life), compared with those who are in a high-quality relationship but all children are living farther away (0.36). The 95% confidence interval of the difference ranges from 0.01 to 0.09. In the male sample, there is no visible difference in predicted probabilities of being very satisfied by living proximity with the same relationship quality.

Similarly, compared with those with all children living farther away, coresidence with high-quality relationships enhances life satisfaction to a greater extent for the rural than the urban sample. Notably, rural elders maintaining a high-quality relationship with children under the same roof report the highest life satisfaction, with a probability of being very satisfied in life being 0.38, which is 6 percentage points higher than those who are also in a high-quality relationship with children but with them living farther away, with the 95% confidence interval in the difference ranging from 0.03 to 0.10. In urban areas, it is the elders who live close to children and maintain a harmonious relationship who are the most satisfied with life. This difference could reflect increased desire for privacy and independence for city dwellers. In urban areas, regardless of living proximity, life satisfaction tends to be lower when parents have a lower relationship quality with their children (0.25 to 0.28 in the probability of being very satisfied). At the same time, lower relationship quality coupled with all children living farther away results in the lowest life satisfaction for rural residents. Rural older adults have a probability of only 0.18 to report being very satisfied with life if all of their children are living farther away and if their relationship quality is lower. This is lower than those who are living with children but with a lower relationship quality (probability of 0.24). The 95% confidence interval of the difference ranges from 0.02 to 0.11. For rural elders, the gap between the bottom group (those with all children living farther away and in lower-quality relationship) and the top group (those with coresident children in a high-quality relationship) is a striking 20 percentage points (0.18 vs. 0.38).

We observe similar patterns for subsamples categorized by marital status and ADL functions. The unmarried (most likely widowed) and ADL-disabled older adults who coreside with their children and have a high-quality relationship report the highest level of life satisfaction. Their advantage in life satisfaction as compared with those with all children living farther away and in a high-quality relationship is much more salient than that in the married and ADL-independent subsamples. For example, in the married sample, proximity hardly makes any difference as long as the relationship quality is high. The difference in the probability of being very satisfied in life between those with coresident children and those with children living farther away is small, holding relationship status constant (0.36 vs. 0.38 for those in a high-quality relationship, and 0.24 vs. 0.24 for those in a lower-quality relationship, see Figure 2c). In contrast, in the unmarried sample, those who are living with children and in a high-quality relationship enjoy an advantage of 8 percentage points higher than with children living farther away and in a high-quality relationship (0.42 vs. 0.34). The 95% confidence interval of the difference ranges from 0.04 to 0.12. We observe the same pattern in the comparison between ADL-independent and ADL-disabled samples, that is, coresidence clearly has added benefit when coupled with a high-quality relationship in the ADL-disabled sample, but it is not the case in the ADL-independent sample.

In sum, we observe a much stronger association of a high-quality intergenerational relation coupled with living proximity among the subsamples of female, rural, unmarried, and ADL-disabled older adults, compared with male, urban, married, and ADL-independent counterparts. The combined effect of being farther away from all children and having a lower-quality relationship is the most detrimental for some of the disadvantaged groups.

## 8. Discussion and conclusion

In her presidential address for the Population Association of America, Judith Seltzer (2019) proposes for the “family uncertainty principle” as families have become more complex and diverse globally and the meanings of family ties and obligations have become blurred. Traditionally family demographers overwhelmingly base their empirical research on the household or treat “family as units”; Seltzer (2019) calls for more focus on “family-as-relationships.” Although the examples she uses in the paper largely reflect recent US family trends, including the growth of stepfamilies and cohabitation, we believe this general principle extends to the East Asian context as well as family structures and family norms are undergoing dramatic transitions.

Our paper has provided clear evidence that to understand the well-being and needs of older adults, it is no longer adequate to focus on their living arrangements, or “family as units.” Instead, it is paramount to study “family as relationships.” While coresidence with adult children used to be the dominant family structure among older adults in China, the majority of older adults are not living with their children, although it is still the most prevalent family type. In addition, a sizable proportion of older adults have adult children living in the same village or neighborhood. At the same time, this decline in coresidence cannot be simply interpreted as a weakening of intergenerational ties. Regardless of where their children live, the majority of the older adults in our sample report close emotional bonds between themselves and their children. Interestingly, our initial finding suggests living proximity per se, that is, whether living with children or having children living nearby does not seem to bring more benefits to older adults’ life satisfaction than having children living farther away. Indeed, our results suggest “proximity without intimacy” is associated with no benefit for subjective well-being. As long as the relationship quality is strong, parents can enjoy a high level of life satisfaction without having children living together with them or close by.

Do our findings suggest that parent–child proximity does not matter? We believe the answer is nuanced. We argue that the intersection of structural solidarity (as indicated by parent–child proximity) and affective solidarity (as indicated by relationship quality) is important, particularly for the vulnerable population. Once taking into account the dynamics of the intergenerational relationship, our findings provide direct evidence that family could be a double-edged sword, that is, coresidence or close-distance living promotes life satisfaction, but only when the intergenerational relationship quality is satisfactory. When the emotional bond is weak or prone to conflicts, close proximity does not help to enhance life satisfaction. Our subsample analyses highlight that a high-quality relationship is particularly important for socioeconomically and physically disadvantaged groups, namely, women, rural, unmarried, and ADL-disabled older adults. Meanwhile, lower-quality relationships paired with all children being farther away further disadvantages these groups, who are most in need of family and social support.

The study is not without limitations. We treat relationship quality and parent–child proximity as independent aspects of intergenerational ties. It is plausible that parents and children who are emotionally close to each other are more likely to choose to live together, while those who have strained relationships are more likely to move apart. The data we use is cross-sectional, so we refrain from making causal inferences. We try to control for selectivity in parent‒child residential patterns by using an instrumental variable approach, but we cannot apply it in all the models due to limits in our instruments (results not shown). With more waves of the data available in the near future, we would be in a better situation to address the issue of endogeneity and unobserved heterogeneity. Our characterization of relationship quality with a dichotomous variable is also a bit crude. Previous literature suggests that both positive and negative components of the parent–child relationship could have different implications for well-being. Another limitation is that because our unit of analysis is the parent, we aggregate information from multiple children to define our measures of parent‒child proximity and relationship quality. By doing so, we may not fully explore the interactions and exchanges between a parent and each of his/her children. For example, for elders who have multiple children, the roles of daughters and sons who both live close by might be different for parents’ well-being. Those who are left behind could be the ones who are engaged in providing instrumental support to the parents while those migrant children could send remittances home and provide economic support. Future analyses could take advantage of multiple parent‒child dyads and take into account different types of intergenerational exchanges.

Despite these limitations, we have made an important contribution to the existing literature. Intergenerational ties transcend the boundary of the household. The beneficial effect of living under the same roof extends to “modified extended family” for which two generations are living close by. Therefore, the swift rise of single-generation households, as observed in China and many other East Asian societies in recent years, should be not simply interpreted as the erosion of intergenerational ties. Moreover, to gain a clear understanding of the consequences of changes in family behavior and obligations in transitional East Asian societies, it is imperative to study the interaction between different dimensions of intergenerational relations. Some Asian societies such as Singapore and Hong Kong have offered preferential treatment for elders and their adult children who opt to live in one unit or two nearby units in the application for public housing. These measures aim to strengthen the structural solidarity, while at the same time, propaganda or policies to consolidate the harmonious and affective relationship between the generations would be of paramount importance.

## Figures and Tables

**Table 1:** Descriptive statistics of covariates in total and subsamples, CLASS 2014

Variable	Total	Male	Female	Urban	Rural	Married	Unmarried	ADL Independent	ADL Disabled
***Dependent variable***									
Life satisfaction (range from 1 to 4)	3.055	3.051	3.058	3.098	2.992	3.061	3.044	3.104	2.958
	[0.879]	[0.867]	[0.891]	[0.863]	[0.900]	[0.867]	[0.903]	[0.849]	[0.930]
***Covariates***									
Age	70.238	69.678	70.747	70.407	69.990	67.948	74.547	68.273	74.172
	[7.991]	[7.873]	[8.063]	[8.064]	[7.877]	[6.876]	[8.162]	[7.042]	[8.319]
Male (Female=0)	0.476			0.472	0.483	0.580	0.282	0.534	0.360
	[0.499]			[0.499]	[0.500]	[0.494]	[0.450]	[0.499]	[0.480]
Urban (Rural=0)	0.595	0.590	0.601			0.610	0.567	0.676	0.435
	[0.491]	[0.492]	[0.490]			[0.488]	[0.496]	[0.468]	[0.496]
Married (Widowed/divorced/unmarried=0)	0.653	0.795	0.524	0.669	0.629			0.733	0.493
	[0.476]	[0.404]	[0.499]	[0.470]	[0.483]			[0.442]	[0.500]
Literate (Illiterate=0)	0.684	0.840	0.543	0.777	0.547	0.768	0.527	0.823	0.407
	[0.465]	[0.367]	[0.498]	[0.416]	[0.498]	[0.422]	[0.499]	[0.382]	[0.491]
Currently working (Not working=0)	0.195	0.251	0.144	0.110	0.320	0.241	0.108	0.209	0.167
	[0.396]	[0.434]	[0.351]	[0.313]	[0.467]	[0.428]	[0.311]	[0.407]	[0.373]
Have public pension (No pension=0)	0.777	0.809	0.748	0.825	0.706	0.797	0.740	0.804	0.723
	[0.416]	[0.393]	[0.434]	[0.380]	[0.456]	[0.402]	[0.439]	[0.397]	[0.448]
MMSE score (range from 0 to 16)	10.231	11.615	8.973	11.523	8.331	11.291	8.238	12.117	6.456
	[5.527]	[4.785]	[5.847]	[5.052]	[5.647]	[5.077]	[5.784]	[4.509]	[5.445]
ADL independent (ADL disabled=0)	0.667	0.748	0.593	0.757	0.535	0.749	0.513		
	[0.471]	[0.434]	[0.491]	[0.429]	[0.499]	[0.434]	[0.500]		
IADL independent (IADL disabled=0)	0.576	0.668	0.493	0.638	0.485	0.656	0.425	0.734	0.259
	[0.494]	[0.471]	[0.500]	[0.481]	[0.500]	[0.475]	[0.494]	[0.442]	[0.438]
The number of children alive	3.032	2.858	3.189	2.747	3.450	2.781	3.504	2.703	3.689
	[1.549]	[1.489]	[1.585]	[1.489]	[1.540]	[1.419]	[1.667]	[1.386]	[1.645]
Any children with post-secondary	0.302	0.324	0.282	0.424	0.122	0.357	0.198	0.387	0.133
	[0.459]	[0.468]	[0.450]	[0.494]	[0.328]	[0.479]	[0.399]	[0.487]	[0.339]
Any children working as managers or professionals	0.254	0.269	0.241	0.326	0.148	0.281	0.203	0.299	0.163
	[0.435]	[0.443]	[0.427]	[0.469]	[0.356]	[0.450]	[0.402]	[0.458]	[0.370]
Observations	10,768	5,128	5,640	6,411	4,357	7,031	3,737	7,181	3,587

*Notes*: The mean values are presented; standard deviation in brackets.

**Table 2:** Distribution of parent–child living proximity paired with relationship quality in total and subsamples (CLASS 2014, N = 10,768)

	Total	Male	Female	Urban	Rural	Married	Unmarried	ADL Independent	ADL Disabled
*Coresidence*	44.2	41.6	46.6	44.4	43.9	38.6	54.9	41.5	49.7
Coresidence with high-quality relation	32.5	30.0	34.8	31.7	33.7	28.2	40.7	30.5	36.6
Coresidence with lower-quality relation	11.7	11.6	11.8	12.7	10.2	10.4	14.1	11.0	13.1
*Children living close by*	18.9	18.1	19.6	14.3	25.6	18.9	18.9	16.4	23.9
Children living close by with high-quality relation	14.3	13.6	14.9	10.8	19.3	14.3	14.2	12.5	17.9
Children living close by with lower-quality relation	4.6	4.5	4.7	3.5	6.3	4.6	4.7	3.9	6.0
*Children all living farther away*	36.9	40.3	33.8	41.3	30.5	42.6	26.3	42.2	26.4
Children all living farther away with high-quality relation	28.8	31.2	26.7	31.6	24.8	33.9	19.4	33.1	20.4
Children all living farther away with lower-quality relation	8.1	9.1	7.1	9.7	5.8	8.8	6.8	9.1	6.0
Observations	10,768	5,128	5,640	6,411	4,357	7,031	3,737	7,181	3,587

**Table 3:** Ordered logit regression models of life satisfaction, proximity of adult children as a key independent variable, CLASS 2014

	Ordered Logit Model			
	Model 1		Model 2	
	Coef.	P-value	Coef.	P-value
Coresidence with children	−0.054	0.172	0.046	0.263
	[0.040]		[0.041]	
Children living close by (ref: Children living farther away)	0.018	0.721	0.066	0.209
	[0.050]		[0.052]	
Age			0.121	0.002
			[0.039]	
Age square			−0.001	0.025
			[0.000]	
Male (Female=0)			−0.207	0.000
			[0.040]	
Urban (Rural=0)			0.002	0.960
			[0.042]	
Married (Widowed/divorced/unmarried=0)			0.055	0.214
			[0.044]	
Literate (Illiterate=0)			−0.001	0.988
			[0.048]	
Currently Working (Not working=0)			0.227	0.000
			[0.050]	
Have public pension (No pension=0)			0.229	0.000
			[0.045]	
MMSE score (range from 0 to 16)			0.029	0.000
			[0.004]	
ADL independent (ADL disabled=0)			0.104	0.031
			[0.048]	
IADL independent (IADL disabled=0)			0.459	0.000
			[0.043]	
Number of children alive			0.058	0.000
			[0.014]	
Any children with post-secondary			0.094	0.044
			[0.047]	
Any children working as managers or professionals			0.287	0.000
			[0.046]	
Intercept1	−2.794	0.000	3.768	0.007
	[0.047]		[1.392]	
Intercept2	−1.156	0.000	5.448	0.000
	[0.032]		[1.392]	
Intercept3	0.569	0.000	7.240	0.000
	[0.031]		[1.392]	
LR chi2	2.948		562.313	
Df	5		19	
BIC	26,156.670		25,727.280	
N	10,768		10,768	

*Note*: Standard errors in brackets.

**Table 4:** Ordered logit regression models of life satisfaction, proximity and parent–adult child relationship quality jointly as key independent variables, CLASS 2014

	Total	Male	Female	Urban	Rural	Married	Unmarried	ADL Indep.	ADL Disabled
Coresidence with high-quality relation	0.731	0.723	0.757	0.572	1.054	0.555	1.065	0.588	1.143
	[0.071]	[0.099]	[0.103]	[0.086]	[0.128]	[0.086]	[0.128]	[0.084]	[0.137]
	*0.000*	*0.000*	*0.000*	*0.000*	*0.000*	*0.000*	*0.000*	*0.000*	*0.000*
Coresidence with lower-quality relation	0.079	−0.039	0.197	−0.085	0.412	−0.040	0.357	−0.079	0.505
	[0.082]	[0.114]	[0.118]	[0.099]	[0.146]	[0.101]	[0.143]	[0.099]	[0.151]
	*0.333*	*0.736*	*0.096*	*0.394*	*0.005*	*0.690*	*0.013*	*0.422*	*0.001*
Children living close by with high-quality relation	0.762	0.700	0.828	0.743	0.936	0.694	0.963	0.708	1.030
	[0.081]	[0.114]	[0.115]	[0.106]	[0.135]	[0.097]	[0.145]	[0.098]	[0.147]
	*0.000*	*0.000*	*0.000*	*0.000*	*0.000*	*0.000*	*0.000*	*0.000*	*0.000*
Children living close by with lower-quality relation	0.048	0.035	0.058	0.050	0.220	0.007	0.192	−0.154	0.519
	[0.106]	[0.153]	[0.148]	[0.147]	[0.163]	[0.130]	[0.185]	[0.135]	[0.179]
	*0.650*	*0.819*	*0.695*	*0.736*	*0.177*	*0.954*	*0.298*	*0.253*	*0.004*
Children all living farther away with high-quality relation	0.657	0.709	0.605	0.608	0.785	0.620	0.723	0.575	0.910
	[0.071]	[0.098]	[0.105]	[0.086]	[0.130]	[0.084]	[0.136]	[0.083]	[0.143]
	*0.000*	*0.000*	*0.000*	*0.000*	*0.000*	*0.000*	*0.000*	*0.000*	*0.000*
Age	0.118	0.147	0.086	0.007	0.294	0.126	0.119	0.041	0.124
	[0.039]	[0.055]	[0.055]	[0.050]	[0.064]	[0.061]	[0.060]	[0.059]	[0.060]
	*0.002*	*0.008*	*0.117*	*0.886*	*0.000*	*0.040*	*0.047*	*0.485*	*0.039*
Age square	−0.001	−0.001	0.000	0.000	−0.002	−0.001	−0.001	0.000	−0.001
	[0.000]	[0.000]	[0.000]	[0.000]	[0.000]	[0.000]	[0.000]	[0.000]	[0.000]
	*0.029*	*0.048*	*0.300*	*0.578*	*0.000*	*0.138*	*0.132*	*0.994*	*0.087*
Male (Female=0)	−0.183			−0.183	−0.199	−0.220	−0.094	−0.221	−0.093
	[0.040]			[0.052]	[0.064]	[0.048]	[0.073]	[0.048]	[0.072]
	*0.000*			*0.000*	*0.002*	*0.000*	*0.197*	*0.000*	*0.197*
Urban (Rural=0)	0.044	0.003	0.089			0.034	0.074	0.018	0.078
	[0.043]	[0.062]	[0.059]			[0.054]	[0.070]	[0.055]	[0.068]
	*0.300*	*0.968*	*0.132*			*0.529*	*0.291*	*0.747*	*0.250*
Married (Widowed/divorced/unmarried=0)	0.039	0.004	0.060	0.076	−0.038			0.060	−0.005
	[0.044]	[0.070]	[0.057]	[0.058]	[0.068]			[0.056]	[0.072]
	*0.376*	*0.953*	*0.293*	*0.189*	*0.580*			*0.278*	*0.942*
Literate (Illiterate=0)	−0.022	−0.054	0.005	−0.108	0.053	0.017	−0.062	0.013	−0.063
	[0.049]	[0.079]	[0.063]	[0.070]	[0.068]	[0.064]	[0.075]	[0.066]	[0.072]
	*0.649*	*0.496*	*0.933*	*0.122*	*0.441*	*0.796*	*0.407*	*0.849*	*0.378*
Currently Working (Not working=0)	0.232	0.223	0.268	0.072	0.348	0.234	0.283	0.149	0.469
	[0.050]	[0.067]	[0.076]	[0.078]	[0.067]	[0.058]	[0.105]	[0.060]	[0.092]
	*0.000*	*0.001*	*0.000*	*0.356*	*0.000*	*0.000*	*0.007*	*0.013*	*0.000*
Have public pension (No pension=0)	0.228	0.251	0.208	0.317	0.142	0.278	0.141	0.247	0.181
	[0.045]	[0.069]	[0.059]	[0.066]	[0.062]	[0.058]	[0.071]	[0.059]	[0.070]
	*0.000*	*0.000*	*0.000*	*0.000*	*0.023*	*0.000*	*0.048*	*0.000*	*0.010*
MMSE score (range from 0 to 16)	0.029	0.039	0.021	0.019	0.039	0.032	0.025	0.029	0.027
	[0.004]	[0.007]	[0.006]	[0.006]	[0.006]	[0.005]	[0.007]	[0.006]	[0.006]
	*0.000*	*0.000*	*0.000*	*0.001*	*0.000*	*0.000*	*0.000*	*0.000*	*0.000*
ADL independent (ADL disabled=0)	0.099	0.038	0.145	0.242	−0.028	0.058	0.181		
	[0.048]	[0.073]	[0.065]	[0.069]	[0.069]	[0.063]	[0.077]		
	*0.041*	*0.602*	*0.025*	*0.000*	*0.681*	*0.352*	*0.018*		
IADL independent (IADL disabled=0)	0.450	0.492	0.412	0.498	0.383	0.502	0.369	0.486	0.362
	[0.043]	[0.064]	[0.058]	[0.058]	[0.064]	[0.053]	[0.072]	[0.053]	[0.074]
	*0.000*	*0.000*	*0.000*	*0.000*	*0.000*	*0.000*	*0.000*	*0.000*	*0.000*
The number of children alive	0.047	0.049	0.043	0.071	0.015	0.049	0.047	0.018	0.082
	[0.014]	[0.021]	[0.019]	[0.019]	[0.021]	[0.020]	[0.021]	[0.019]	[0.021]
	*0.001*	*0.024*	*0.023*	*0.000*	*0.494*	*0.012*	*0.023*	*0.347*	*0.000*
Any children with post-secondary	0.064	0.116	0.014	0.016	0.245	0.070	0.036	0.043	0.069
	[0.047]	[0.067]	[0.066]	[0.054]	[0.098]	[0.055]	[0.088]	[0.053]	[0.102]
	*0.173*	*0.083*	*0.832*	*0.771*	*0.012*	*0.205*	*0.680*	*0.420*	*0.497*
Any children working as managers or professionals	0.289	0.255	0.324	0.268	0.340	0.284	0.301	0.307	0.273
	[0.046]	[0.066]	[0.065]	[0.054]	[0.088]	[0.055]	[0.084]	[0.053]	[0.093]
	*0.000*	*0.000*	*0.000*	*0.000*	*0.000*	*0.000*	*0.000*	*0.000*	*0.003*
Intercept1	4.049	5.379	2.838	0.000	10.364	4.231	4.315	1.184	4.606
	[1.396]	[1.988]	[1.973]	[1.788]	[2.306]	[2.154]	[2.239]	[2.087]	[2.244]
	*0.004*	*0.007*	*0.150*	*1.000*	*0.000*	*0.049*	*0.054*	*0.570*	*0.040*
Intercept2	5.752	7.146	4.491	1.770	12.002	5.958	5.990	3.012	6.160
	[1.396]	[1.988]	[1.973]	[1.787]	[2.307]	[2.154]	[2.239]	[2.086]	[2.244]
	*0.000*	*0.000*	*0.023*	*0.322*	*0.000*	*0.006*	*0.007*	*0.149*	*0.006*
Intercept	7.578	9.055	6.249	3.616	13.822	7.856	7.696	4.907	7.871
	[1.397]	[1.990]	[1.974]	[1.788]	[2.310]	[2.155]	[2.241]	[2.087]	[2.246]
	*0.000*	*0.000*	*0.002*	*0.043*	*0.000*	*0.000*	*0.001*	*0.019*	*0.000*
Df	22	21	21	21	21	21	21	21	21
BIC	25507.610	12082.760	13551.550	14960.840	10626.940	16524.810	9089.865	16594.090	8991.924
LR chi2	809.842	455.373	375.912	497.199	349.659	570.409	273.190	553.661	252.512
N	10768	5128	5640	6411	4357	7031	3737	7181	3587

*Note*: Standard errors in parentheses, P-value in italics.

## Footnotes

4 UN (2017) reports the share of households that include both a child under age 15 and an older person aged 60 or over. Most of these households are multigenerational households, and a tiny share are skipped-generation households.
